# Expanding the Averievite Family, (*MX*)Cu_5_O_2_(*T*^5+^O_4_)_2_ (*T*^5+^ = P, V; *M* = K, Rb, Cs, Cu; *X* = Cl, Br): Synthesis and Single-Crystal X-ray Diffraction Study

**DOI:** 10.3390/molecules26071833

**Published:** 2021-03-24

**Authors:** Ilya V. Kornyakov, Victoria A. Vladimirova, Oleg I. Siidra, Sergey V. Krivovichev

**Affiliations:** 1 Department of Crystallography, Institute of Earth Sciences, St. Petersburg State University, University Emb. 7/9, 199034 Saint-Petersburg, Russia; vladimirovav.sbk.1998@yandex.ru (V.A.V.); o.siidra@spbu.ru (O.I.S.); s.krivovichev@ksc.ru (S.V.K.); 2 Laboratory of Nature-I and Pired Technologies and Environmental Safety of the Arctic, Kola Science Centre, Russian Academy of Sciences, Fersmana 14, 184209 Apatity, Russia; 3 Institute of Silicate Chemistry, Russian Academy of Sciences, Adm. Makarova emb. 2, 199034 St. Petersburg, Russia; 4 Nanomaterials Research Center, Federal Research Center–Kola Science Center, Russian Academy of Sciences, Fersmana Str. 14, 184209 Apatity, Russia

**Keywords:** averievite, synthesis, chemical vapor transport, X-ray diffraction, crystal structure, kagome lattice, oxocentered tetrahedra

## Abstract

Averievite-type compounds with the general formula (*MX*)[Cu_5_O_2_(*T*O_4_)], where *M* = alkali metal, *X* = halogen and *T* = P, V, have been synthesized by crystallization from gases and structurally characterized for six different compositions: **1** (*M* = Cs; *X* = Cl; *T* = P), **2** (*M* = Cs; *X* = Cl; *T* = V), **3** (*M* = Rb; *X* = Cl; *T* = P), **4** (*M* = K; *X* = Br; *T* = P), **5** (*M* = K; *X* = Cl; *T* = P) and **6** (*M* = Cu; *X* = Cl; *T* = V). The crystal structures of the compounds are based upon the same structural unit, the layer consisting of a kagome lattice of Cu^2+^ ions and are composed from corner-sharing (OCu_4_) anion-centered tetrahedra. Each tetrahedron shares common corners with three neighboring tetrahedra, forming hexagonal rings, linked into the two-dimensional [O_2_Cu_5_]^6+^ sheets parallel to (001). The layers are interlinked by (*T*^5+^O_4_) tetrahedra (*T*^5+^ = V, P) attached to the bases of the oxocentered tetrahedra in a “face-to-face” manner. The resulting electroneutral 3D framework {[O_2_Cu_5_](*T*^5+^O_4_)_2_}^0^ possesses channels occupied by monovalent metal cations *M*^+^ and halide ions *X*^−^. The halide ions are located at the centers of the hexagonal rings of the kagome nets, whereas the metal cations are in the interlayer space. There are at least four different structure types of the averievite-type compounds: the *P*-3*m*1 archetype, the 2 × 2 × 1 superstructure with the *P*-3 space group, the monoclinically distorted 1 × 1 × 2 superstructure with the *C*2/*c* symmetry and the low-temperature *P*2_1_/*c* superstructure with a doubled unit cell relative to the high-temperature archetype. The formation of a particular structure type is controlled by the interplay of the chemical composition and temperature. Changing the chemical composition may lead to modification of the structure type, which opens up the possibility to tune the geometrical parameters of the kagome net of Cu^2+^ ions.

## 1. Introduction

Mineralogical information provides an important resource for the design of novel materials with a unique structural architecture and physical properties [1,2,3]. Of particular interest are minerals containing transition metal cations such as Cu^2+^ that form various cation arrays with interesting magnetic behavior. The kagome lattice consisting of corner-sharing Cu_3_ triangles forming a 2D net with regular hexagonal rings is probably the most famous example—such an array is geometrically frustrated, and this feature is responsible for the spin liquid behavior first demonstrated for synthetic herbertsmithite and later observed for other synthetic mineral analogues [4,5,6,7,8,9,10,11]. In order to exhibit a spin liquid property, the kagome array should possess an ideal trigonal (or hexagonal) symmetry, which is absent in most minerals with the kagome arrangement of Cu^2+^ magnetic ions. In addition to the herbertsmithite–paratacamite family mentioned above, regular symmetry has been observed for averievite, another rare copper mineral with a unique mineralogical origin that deserves particular attention.

In 1975–1976, the Kamchatka Peninsula (Russian Far East) experienced one of the largest basalt volcanic eruptions in modern history, termed the Great Tolbachik Fissure Eruption. The extensive post-eruption activity resulted in the formation of fumarolic fields with extensive and structurally and chemically diverse mineralization [12,13], including associations of Cu^2+^ minerals, most of which still have no synthetic counterparts [8,14,15]. A particularly interesting class of fumarolic Cu minerals is those containing ”additional” oxygen atoms, i.e., atoms that do not participate in strongly bonded “acid residues” (such as sulfate, selenate, arsenate, chlorides, fluorides, etc.) and form anion-centered (OCu_4_) tetrahedra [16].

Averievite, Cu_5_O_2_(VO_4_)_2_·*n*MCl*_x_* (M = Cu^+^, Cs, Rb, K), was found as a product of the post-eruption volcanic activity of the Great Tolbachik Fissure Eruption in 1997 [17,18]. Originally, the crystal structure of the mineral had been solved and refined in the *P*$\bar{3}$ space group, but a later study showed that the *P*$\bar{3}$*m*1 space group is more correct [19]. The Cu^2+^ ions in the structure are arranged in a perfect kagome array, which prompted its consideration as a possible candidate for quantum spin liquid behavior [20]. Computational theoretical studies of the averievite structure have been carried out by Volkova and Marinin [21]. More recent experimental studies showed that the vanadate ion in averievite can be replaced by phosphate [22], which results in the increasing chemical pressure influencing the electronic and magnetic properties of the material [23]. Previous crystal structure studies of synthetic averievite [20,22,23] were conducted by means of Rietveld refinement of its powdered samples.

Herein, we report on the synthesis and crystal structures of averievite-type compounds and the single-crystal X-ray diffraction analysis of their structures. We demonstrate that the interplay between the chemical composition and symmetry of synthetic averievites essentially affects the resulting structure type and that there are at least four different structure types of materials with the averievite structure topology.

## 2. Materials and Methods

### 2.1. Synthesis

Single crystals of **1**, **3**, **4**, **5**, and **6** were prepared via crystallization from gaseous phase, previously employed for the modeling of fumarolic mineral formation in [24,25]. Stoichiometric amounts of copper oxide (CuO, 99%, Vekton, St. Petersburg, Russia), copper pyrophosphate (Cu_2_P_2_O_7_, 99%, Vekton) and alkali metal halides (CsCl for **1**, 99%, Vekton; RbCl for **3**, 99%, Vekton; KBr for **4**, 99%, Vekton; KCl for **5**, 99%, Vekton) were ground in an agate mortar. The mixtures were placed in a fused silica ampule (ca. 13 cm), which was evacuated to ~10^−2^ mbar, sealed and placed horizontally in a furnace and heated to 800 °C over a period of 6 h. After 2 days, the furnace was cooled to 350 °C over a period of 72 h and switched off. The resulting samples contained single crystals of title compounds and copper oxide. Attempts to synthesize phosphate-bearing compounds of the averievite family containing other alkali metal halides (NaCl, Cu^+^Cl_2_) were unsuccessful.

Single crystals of **2** were prepared using the chemical vapor transport reaction technique [24]. Copper oxide (CuO, 99%, Vekton), vanadium oxide (V_2_O_5_, 99%, Vekton) and cesium chloride (CsCl, 99%, Vekton) in a 1:1:2 molar ratio were ground in an agate mortar and the resulting mixture was heated at 250 °C for 16 h in air. The sample was further loaded into a fused silica ampule (ca. 10 cm), which was evacuated to 10^−2^ mbar before sealing. The ampule was placed horizontally into a two-zone furnace and heated to 650 °C within 4 h. The temperature gradient between the source and deposition zones of the ampule was ~50 °C. After 2 days, the ampule was cooled to 250 °C over a period of 72 h and then the furnace was switched off. Hexagonal black crystals of **2** were found in the deposition zone of the ampule in association with green crystals of [CsCl]_2_Cu(VO_3_)_2_ [26].

**Figure 1:**
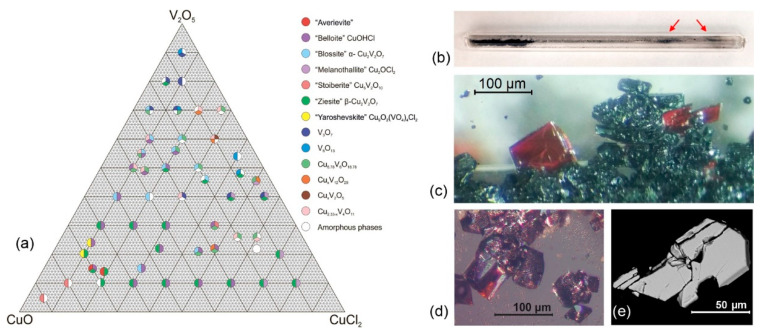
(**a**) Experimental crystallization diagram of the CuO-V_2_O_5_-CuCl_2_ system at 600 °C. Sectorial color points indicate the formation of different phases (identified by single-crystal X-ray diffraction) listed to the right. Corresponding mineral names are given in quotation marks. (**b**) Sealed quartz ampule with red arrows pointing at the deposition zone with averievite Cu_5_O_2_(VO_4_)_2_·CuCl crystals. (**c**) Red averievite crystals in association with ziesite β-Cu_2_V_2_O_7_. (**d**) Selected averievite crystals under optical microscope. (**e**) Scanning electron microscopy (SEM) image of averievite crystal.

We investigated the phase formation in the ternary system CuO-V_2_O_5_-CuCl_2_ (Figure 1) without the presence of alkali metal cations. The formation of crystals of averievite, Cu_5_O_2_(VO_4_)_2_·CuCl, was observed for several ratios (5:1:1 and 4.78:1:1.36) of the starting reagents. By analogy with the natural copper-vanadate-chlorides that form upon high-temperature exhalative processes occurring in the fumarole fields of the Tolbachik volcano, crystalline samples of various mineral analogues (Figure 1) were obtained by the gas transport reactions from the mixtures containing different ratios of CuO (99.995%, Sigma-Aldrich, St. Louis, MO, U.S.A.), V_2_O_5_ (Sigma-Aldrich, 99.6%) and CuCl_2_ (Sigma-Aldrich, 99%). All of the reagents were pre-dried at 100 °C for 5 h and further rapidly mixed and ground in an agate mortar in air for approx. 5 min. The reaction mixtures were loaded into quartz ampules (ca. 15 × 0.9 cm), which were evacuated (10^−2^ mbar) and further sealed. The ampules were placed horizontally in a tubular furnace (Nabertherm) and heated up to 600 °C at a rate of 60 °C/hour and further held at this temperature for 6 h. The temperature gradient between the source (hot) and deposition (cold) zones of the tube in the furnace was about 50 °C. Note that the most of the mineral phases containing additional oxygen atoms (yaroshevskite Cu_9_O_2_(VO_4_)_4_Cl_2_ [25,27] and stoiberite Cu_5_O_2_(VO_4_)_2_ [28]) crystallize in the region where copper oxide prevails in the initial charge. Note the presence of belloite CuOHCl in a number of syntheses indicated in the triangular diagram (Figure 1). This observation was the result of the partial hydration of the initial mixture of reagents for a short period of time in the air before filling the quartz ampules. Note, also, the partial reduction of copper (Cu^2+^→Cu^+^) and vanadium (V^5+^→V^4+^) in many syntheses listed in Figure 1. The partial reduction of copper in chloride complexes is also responsible for the formation of averievite Cu_5_O_2_(VO_4_)_2_·CuCl.

All the studied compounds were stable in air and their crystals did not experience any deterioration in the course of the experimental studies.

### 2.2. Single-Crystal X-ray Diffraction Study

Single crystals of **1**–**6** were selected for data collection under an optical microscope, coated in oil-based cryoprotectant and mounted on cryoloops. Diffraction data for **1**–**4** and **6** were collected using a Bruker APEX II DUO X-ray diffractometer (Bruker Co., Billerica, MA, U.S.A.) operated with monochromated microfocus MoKα tube (*λ* = 0.71073 Å) at 50 kV and 0.6 mA and equipped with a CCD APEX II detector. Exposures varied from 30 to 120 s per frame. CrysAlisPro software [29] was used for integration and correction of diffraction data for polarization, background and Lorentz effects as well as for an empirical absorption correction based on spherical harmonics implemented in the SCALE3 ABSPACK algorithm. The unit cell parameters (Table 1) were refined using the least-squares technique. The structures were solved by a dual-space algorithm and refined using the SHELX programs [30,31] incorporated in the OLEX2 program package [32]. The final models include coordinates and anisotropic displacement parameters.

Diffraction data for **5** were collected using a Rigaku XtaLAB Synergy S X-ray diffractometer (Rigaku Co., Tokyo, Japan) operated with a monochromated microfocus MoKα tube (*λ* = 0.71073 Å) at 50 kV and 1.0 mA and equipped with a CCD HyPix 6000 detector. The frame width was 1.0° in *ω*, and there was a 200-s count time for each frame. CrysAlisPro software [29] was used for integration and correction of diffraction data for polarization, background and Lorentz effects as well as for an empirical absorption correction based on spherical harmonics implemented in the SCALE3 ABSPACK algorithm. The unit cell parameters (Table 1) were refined using the least-squares technique. The structure was solved by a dual-space algorithm and refined using the SHELX programs [30,31] incorporated in the OLEX2 program package [32]. The final models include coordinates and anisotropic displacement parameters. Table A1 provides selected interatomic distances in the studied compounds.

**Figure 2:**
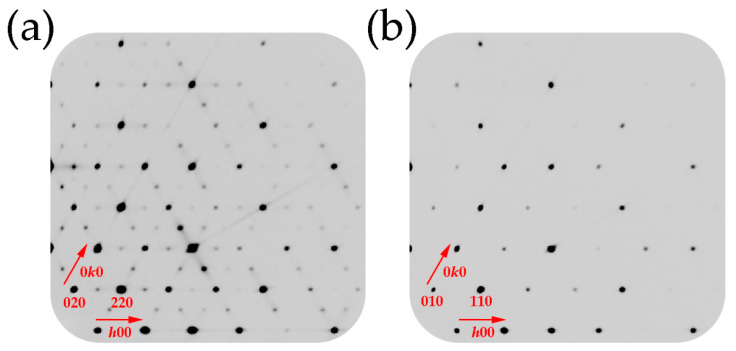
(**a**) A fragment of a precession image of the *hk*0 reciprocal lattice plane of compound **2**; (**b**) a fragment of a precession image of the *hk*0 reciprocal lattice plane of compound **1**.

During the collection of diffraction data, it was found that the *a* and *b* unit cell parameters of compound **2** are doubled (12.725 Å) compared to the reported averievite structure (6.378 Å) [18]. A careful inspection of the reciprocal space confirmed the presence of superlattice reflections (Figure 2). The structure was solved and refined in the *P*$\bar{3}$*m*1 space group with the final *R*_1_ factor converged to 0.10. In order to improve final convergence factors, the structure was solved and refined once again in the *P*$\bar{1}$ space group, resulting in the spectacular improvement of the *R*_1_ value to 0.04. The ADDSYM program within the PLATON software package [33] was applied to the obtained the structural model in the *P*$\bar{3}$ space group. Further attempts to increase the symmetry led to *a* and *b* unit cell parameters of 6.362 Å and *P*$\bar{3}$*m*1 space group. The final structural model was refined in the *P*$\bar{3}$ space group with an *R*_1_ value of 0.03.

All the tested single crystals of **3**, **4** and **5** were found to be twinned. Inspection of the full diffraction data of both compounds by means of the CrysAlisPro software revealed the presence of non-merohedral twinning, which was processed during data integration with the simultaneous creation of the HKLF5-type reflection files. The final structural models of **3** and **5** were refined on the basis of a single twin domain, whereas the final model of **4** was refined on the basis of two domains with the refined twin ratio of 0.730(2):0.270(2).

Crystallographic Information Files (CIFs) for the studied compounds are given as Supplementary Material.

## 3. Results

**Figure 3:**
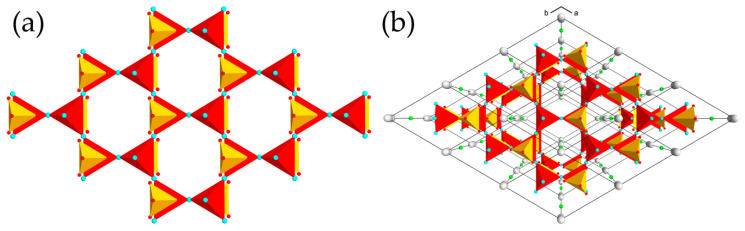
(**a**) The [O_2_Cu_5_]^6+^ layer of oxocentered tetrahedra (red) with (PO_4_)^3−^ tetrahedra (yellow) attached in a “face-to-face” fashion in the crystal structure of **1**; (**b**) the crystal structure of **1** projected along the *c* axis. Legend: Cu, Cs and Cl atoms are shown as cyan, gray and green spheres, respectively.

The crystal structures of the averievite-type compounds are based upon the same structural unit—the layer consisting of kagome lattice of Cu^2+^ ions. The layer is composed from corner-sharing (OCu_4_) anion-centered tetrahedra. Each tetrahedron shares common corners with three neighboring tetrahedra, forming hexagonal rings, linked into the two-dimensional [O_2_Cu_5_]^6+^ sheets parallel to (001). The tetrahedral corners that do not participate in the intralayer linkage are oriented alternately either up (**U**) or down (**D**) relative to the plane of the layer, giving **UDUDUD** hexagonal rings. The layers are interlinked by (*T*^5+^O_4_) tetrahedra (*T*^5+^ = V, P) attached to the bases of the oxocentered tetrahedra in a “face-to-face” manner [34,35] (Figure 3a). The resulting electroneutral 3D framework {[O_2_Cu_5_](*T*^5+^O_4_)_2_}^0^ possesses channels occupied by monovalent metal cations (Cu^+^, Na, K, Rb, Cs) and halide ions (Cl, Br, I) (Figure 3b). The halide ions are located at the centers of the hexagonal rings of the kagome nets, whereas the metal cations are in the interlayer space.

**Figure 4:**
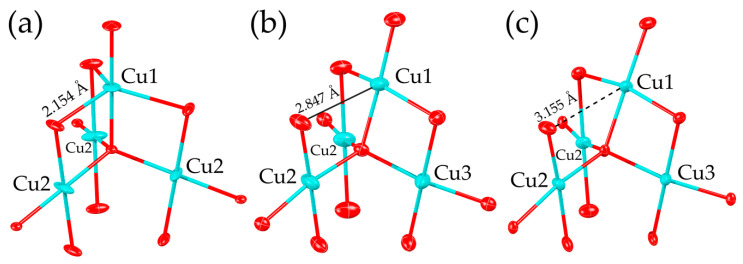
The (OCu_4_) tetrahedron and the first coordination spheres of copper atoms in the crystal structures of **1** (**a**), **3** (**b**) and **4** (**c**). The thermal ellipsoids are drawn at the 50% probability level. Legend: as in Figure 1.

A perfect example of the averievite-type structure is the compound **1**, (CsCl)Cu_5_O_2_(PO_4_)_2_, which can be considered as an archetype structure, i.e., the structure with the maximal possible symmetry. It contains two symmetrically distinct copper atoms. The Cu1 atom is coordinated by five oxygen atoms in a triangular bipyramidal arrangement (Figure 4). The apical Cu1–O_ap_ bond lengths are equal to 1.840 and 1.863 Å, whereas the equatorial Cu1–O_eq_ bond lengths are equal to 2.154 (×3) Å. The (Cu1O_5_) triangular bipyramid is almost perfect with O_eq_–Cu1–O_eq_ and O_ap_–Cu1–O_ap_ angles of ~117° and 180°, respectively. The slight distortion is due to the shifting of the Cu^2+^ ion from the equatorial plane toward one of the apical vertices, which results in a decrease in the O_eq_–Cu1–O_eq_ angle from the ideal 120° to ~117°. The Cu2 atom is at the center of the (Cu2O_4_) planar square, with Cu2–O bond distances equal to 2.025 (×2) and 1.854 (×2) Å and O–Cu2–O angles of ~96° (×2) and ~84° (×2). Both Cu atoms are bonded to the additional oxygen atom O3, forming one O3–Cu1 and three O3–Cu2 bonds. The bond valence sum for the O3 atom is noticeably higher than 2 v.u. (valence units) and is equal to 2.47 v.u. due to the relatively short O3–Cu bond lengths of 1.854 (×3) and 1.863 Å. Special attention should be paid to the disorder of the Cs^+^ cations. Winiarski et al. [21] described the disordered model of Cs^+^ as a double well-type potential within voids of the electroneutral framework. Our single-crystal structure refinement suggests a slightly different arrangement of the disordered Cs^+^ positions. The Cs1A site is located at the 1a Wyckoff site, whereas Cs1B occupies the 2c Wyckoff site. During the refinement, the site occupancy factors (SOFs) for both positions were set free to obtain the correct occupancies. The final SOF values are equal to 0.60 and 0.20 for the Cs1A and Cs1B positions, respectively, which provides the total amount of 1 Cs per formula unit. The distance between the Cs positions is 0.43 Å.

The unit cell of the structure **2**, (CsCl)Cu_5_O_2_(VO_4_)_2_, is four times larger than the unit cell of **1**, which results in doubling the amount of symmetrically distinct positions of Cu^2+^, *T*^5+^, Cs^+^, Cl^−^ and additional O atoms. However, the general structural architecture remains the same and distinguishes from the archetype in details only. Except for the obvious substitution of P^5+^ by V^5+^, the first notable difference is the disorder model of the Cs^+^ cations. There are two symmetrically distinct Cs sites, both with partial occupancy. The Cs1 position is located at the 2c Wyckoff site with the occupancy of 0.5, and the Cs1–Cs1 distance is equal to 0.832 Å. The Cs2 site is split into two symmetrically distinct Cs2A and Cs2B positions located at the 6g Wyckoff sites, with SOF values equal to 0.28 and 0.22, respectively. The bond-vance sum (BVS) values for the additional oxygen atoms are equal to 2.30 and 2.31 v.u. for the O7 and O8 atoms, respectively. It is noteworthy that these BVS values are in good agreement with the values calculated on the basis of the original mineral structure and the structure reported by Botana et al. [19,20].

**Figure 5:**
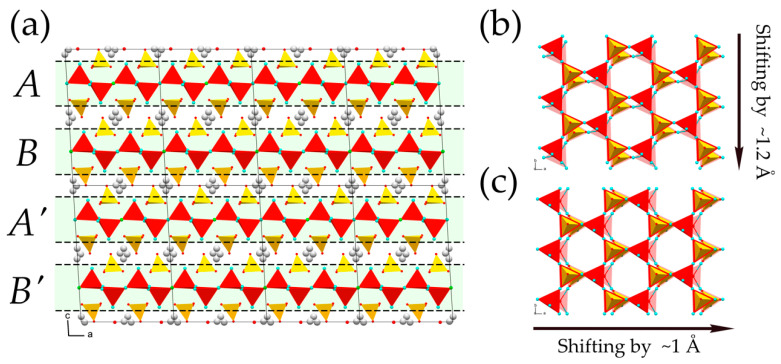
(**a**) The lateral view of the crystal structure of **3** with particular emphasis on the layered motif of the structure; (**b**) the *A* and *B* layers projected along the *c* axis; (**c**) the *A* and *A*′ layers projected along the *c* axis. Dashed lines and light green shading in (**a**) highlight the [O_2_Cu_5_]^6+^ layers. Legend: as in Figure 4.

The structure of **3**, (RbCl)Cu_5_O_2_(PO_4_)_2_, demonstrates a significant change in the averievite-type structural architecture. First of all, the substitution of Cs by Rb leads to significant changes in the arrangement of oxygen atoms around the Cu1 site that change its coordination from triangular bipyramidal to square pyramidal. One of the equatorial oxygen atoms drifts away from the Cu1 site, forming an elongated apical bond of a square pyramid with a distance equal to 2.847 Å (Figure 4b). Two remaining oxygen atoms approach the Cu1 atom, leading to the formation of a square pyramid with Cu1–O_eq_ equatorial bond distances varying from 1.854 to 1.992 Å. Two other symmetrically distinct Cu sites possess a square planar coordination with slight O–Cu–O angular distortions and bond length deviations (1.869–2.036 Å). The geometry of the oxocentered (O5Cu_4_) tetrahedron changes along with the Cu1 coordination; the BVS is, again, higher than the formal oxidation state and is equal to 2.31 v.u. Despite the geometrical changes in the oxocentered tetrahedra, the geometry of the kagome plane remains the same. However, the arrangement of the [O_2_Cu_5_]^6+^ layers relative to each other differs from that observed in the archetype. The unit cell of **3** contains two [O_2_Cu_5_]^6+^ layers, denoted as *A* and *B* in Figure 5a. The layer *B* is shifted by ca. 1.2 Å in the [010] direction relative to the layer *A* (Figure 5b). The next layer *A*′ is shifted by ca. 1 Å in the [100] direction relative to the previous layer *A* (Figure 5b), etc. Such an arrangement affects the geometry of the framework channels, which become inclined relative to the plane of the [O_2_Cu_5_]^6+^ layer by ~3°. Analogous to the archetype, the Cl^−^ ions are at the center of the hexagonal ring of the kagome net, and the Rb site is splitted into two symmetrically distinct positions with SOF values of 0.25.

The deformation of the averievite-type structure is enhanced by the intercalation of K^+^ and *X*^−^ (*X*^−^ = Cl, Br) ions into the channels, as exemplified by the crystal structures of **4** and **5**, (KBr)Cu_5_O_2_(PO_4_)_2_ and (KCl)Cu_5_O_2_(PO_4_)_2_, respectively. In these compounds, the Cu1O_5_ square pyramid is transformed into the Cu1O_4_ planar square due to the elongation of the Cu1-O bond to 3.155 and 3.088 Å for **4** and **5**, respectively. The other Cu atoms are in a square planar coordination. Another important feature of **4** and **5** is the “relaxation” of the (O5Cu_4_) tetrahedron, manifesting in the decrease in the BVS value of the O5 atom to 2.19 and 2.21 v.u., respectively. The [O_2_Cu_5_]^6+^ layers are shifted in the same manner as in the structure of **3**: the neighboring *A* and *B* layers in **4** and **5** are shifted by ca. 1.8 Å relative to each other, whereas the *A* and *A*′ layers are shifted by ca. 1.5 Å. As in the structure of **3**, the K-*X*-bearing channels are inclined to the plane of the [O_2_Cu_5_]^6+^ layer by ~5°. The K1 position is split by an inversion center into two sites with SOF values equal to 0.5.

The structure of **6** is similar to that of **1** described above. It is characterized by the disorder of several atomic sites. The Cu2 site is split over the two sites, Cu2 and Cu2A. The intrachannel positions in **6** are strongly disordered. The electroneutrality of the intrachannel copper–chloride complexes implies the presence of Cu^2+^ cations in addition to monovalent copper cations. There is a possibility of significant ionic mobility with increasing temperature in the voids of the metal oxide framework, which might act as an “electronic reservoir”.

## 4. Discussion

The results of the current study as well as previous results on averievite-type materials [20,22,23] demonstrate that the averievite structural topology is rather flexible, leading to several different structure types depending upon the chemical composition. At least four different structure types are possible: the *P*-3*m*1 archetype, the 2 × 2 × 1 superstructure with the *P*-3 space group, the monoclinically distorted 1 × 1 × 2 superstructure with the *C*2/*c* symmetry and the low-temperature *P*2_1_/*c* superstructure with a doubled unit cell relative to the high-temperature archetype. It seems likely that the formation of a particular structure type is controlled by the interplay of the chemical composition. If the general formula of an averievite-type material is written as (*MX*)[Cu_5_O_2_(*T*O_4_)], where *M* = alkali metal, *X* = halogen and *T* = P, V, then each compound can be associated with the point in the chemical *M-X-T* space separated into regions of the corresponding structure type. Changing the chemical composition may lead to modification of the structure type, which opens the possibility to tune the geometrical parameters of the kagome net of Cu^2+^ ions.

**Figure 6:**
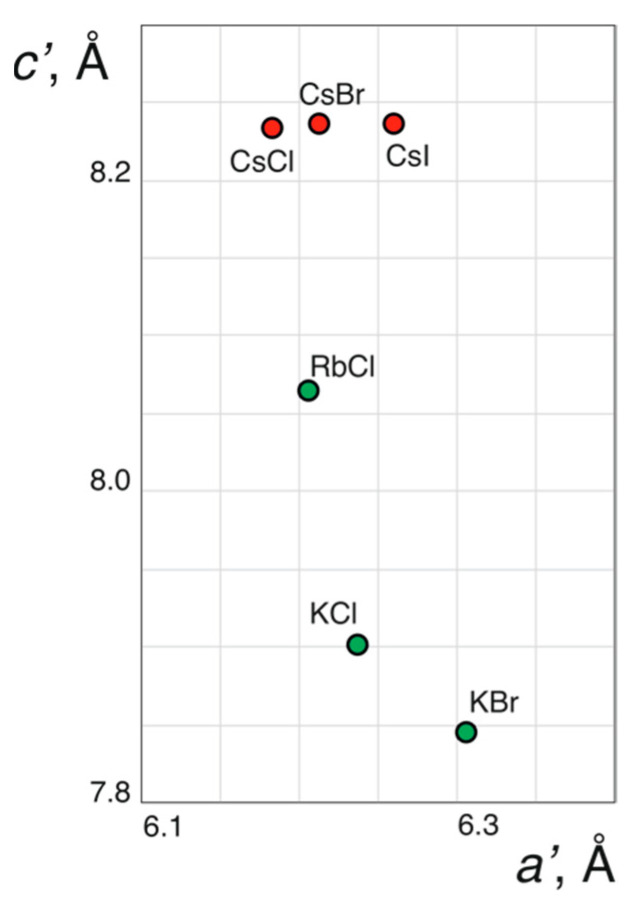
The dependence of the *a*′ and *c*′ parameters of the pseudo-trigonal sub-cell of the phosphate members of the averievite family (coinciding with the *a* and *c* parameters for trigonal members). The green and red colors correspond to monoclinic and trigonal structures, respectively.

**Figure 7:**
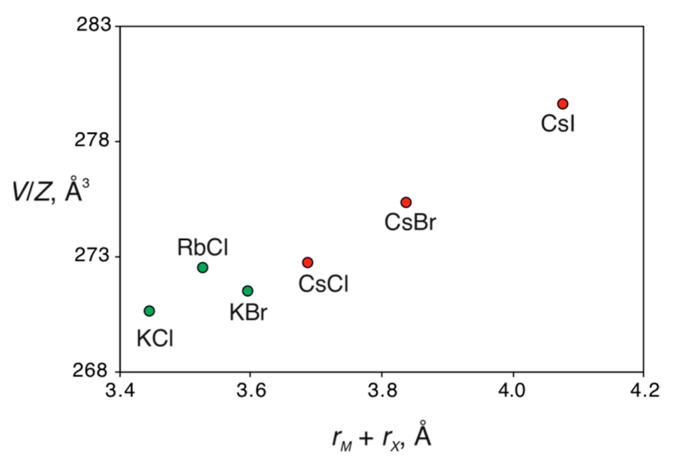
The dependence of the unit cell volume per formula unit, *V*/*Z*, from the sum of ionic radii of *M*^+^ and *X*^−^ ions for the phosphate members of the averievite family. The green and red colors correspond to monoclinic and trigonal structures, respectively.

Analysis of the crystal structures of the phosphate members of the family indicates that the effect of the size of the halide and alkali metal ions on the unit cell dimensions is defined by their structural functions. Large halide ions correspond to the expanded kagome layers with enlarged *a* parameters, owing to their location at the center of the hexagonal rings. In contrast, alkali ions are in the cages in between the layers, and an increase in their size results in the expansion of the interlayer distance (manifested in the enlarged *c* parameters) (Figure 6). Figure 7 shows the dependence of the unit cell volume per formula unit, *V*/*Z*, from the sum of ionic radii of *M*^+^ and *X*^−^ ions (taken for coordination numbers of 12 and 6, respectively [36]). It is remarkable that the trigonal structure type is observed for Cs-bearing phosphate members only, whereas all K- and Rb-bearing compounds are monoclinic. For the Cs- and K-phosphate averievites, the *V*/*Z* parameter changes linearly with the increasing size of the *X*^−^ anion. The RbCl member violates the linear trend, the reason for which is unclear. Botana et al. [20] reported on the synthesis of an averievite-type solid with the chemical formula (CsCl)Cu_3_Zn_2_O_2_(VO_4_)_2_, which shows that the chemical diversity of the family is not restricted to the *M-X-T* space but can also be extended to other metals in the Cu sites.

The nature of the alkali metal cation in the structures of the averievite-type phosphates also affects the geometry of (OCu_4_) tetrahedra. The Cu–O–Cu angles within the kagome plane in the structure of **1** are equal to 113° (×3), whereas the same angles in the structure of **3** are 117.3°, 111.7° and 109.9°. The respective angles in the structure of **4** are equal to 118.4°, 112.0° and 108.0°. The most significant change is observed for the Cu–O–Cu angles out of the kagome plane; the Cu2–O–Cu2 angle in the structure of **1** is 105.7° (×3), whereas the respective angles in the structure of **3** are equal to 119.6°, 99.6° and 98.3°, reflecting the rearrangement of Cu1 coordination geometry, which is enhanced in the structure of **4** with angles of 126.3°, 96.7° and 95.3°. The variation of Cu–O–Cu angles could potentially lead to significant changes in antiferromagnetic coupling within the kagome plane [37,38].

The difference between the structure types can be evaluated using information-based measures of structural complexity [39,40]. The archetype structure of **1** is the simplest one (2.760 bit/atom and 52.446 bit/cell), whereas its 2 × 2 × 1 superstructure (**2**) is the most complex one (3.977 bit/atom and 302.235 bit/cell). The monoclinically distorted archetype with the doubled *c* parameter (as observed in **3**, **4** and **5**) has intermediate complexity measures (3.406 bit/atom and 129.421 bit/cell). The low-temperature *P*2_1_/*c* structure observed for the vanadate members of the family [20,22] is more complex (3.422 bit/atom and 136.877 bit/cell) than the high-temperature polymorph (2.760 bit/atom and 52.446 bit/cell), which agrees well with the overall trend of increasing complexity with decreasing temperature [39].

## Figures and Tables

**Table 1:** Crystallographic data and refinement parameters for **1**–**6**.

Compound	1	2	3	4	5	6
Formula	(CsCl)Cu_5_O_2_(PO_4_)_2_	(CsCl)Cu_5_O_2_(VO_4_)_2_	(RbCl)Cu_5_O_2_(PO_4_)_2_	(KBr)Cu_5_O_2_(PO_4_)_2_	(KCl)Cu_5_O_2_(PO_4_)_2_	(CuCl)Cu_5_O_2_(VO_4_)_2_
Space Group	*P*$\bar{3}$*m*1	*P* $\bar{3}$	*C*2/*c*	*C*2/*c*	*C*2/*c*	*P*$\bar{3}$*m*1
*a*, Å	6.1842(1)	12.7249(4)	10.8869(4)	10.9738(5)	10.9808(10)	6.406(4)
*b*, Å	6.1842(1)	12.7249(4)	6.2074(3)	6.3067(3)	6.2384(5)	6.406(4)
*c*, Å	8.2333(2)	8.3767(2)	16.1562(7)	15.7610(5)	15.8684(14)	8.403(5)
*β*, °	90	90	93.377(4)	95.424(4)	95.227(8)	90
*V*, Å^3^	272.691(11)	1174.66(8)	1089.93(8)	1085.91(8)	1082.51(16)	298.6(4)
*μ*, mm^−1^	13.460	13.733	14.615	14.032	10.632	14.178
*Z*	1	4	4	4	4	1
*D*_calc_, g/cm^3^	4.311	4.233	4.026	4.029	3.769	4.324
Color	Light yellowish green	Black	Dark green	Dark green	Dark green	Black
Total reflections	2219	6203	11646	3738	5375	7232
Unique reflections	269	1810	2632	3738	1218	643
Reflection with |*F*_o_| ≥ 4σ*_F_*	267	1371	2214	3572	1077	578
Angle range 2*θ*, °, MoKα	7.61 to 54.998	3.696 to 54.934	7.5 to 74.606	5.192 to 59.992	7.454 to 54.994	4.85 to 75.332
*R*_int_, *R*_σ_	0.0086, 0.0032	0.0223, 0.0135	0.0527, 0.0307	Merged ^1^, 0.0091	0.0802, 0.0506	0.0315, 0.0145
*R*_1_, *wR*_2_ (|*F*_o_| ≥ 4σ*_F_*)	0.0153, 0.0365	0.0332, 0.0944	0.0562, 0.1467	0.0552, 0.1395	0.0626, 0.1539	0.0326, 0.0784
*R*_1_, *wR*_2_ (all data)	0.0154, 0.0365	0.0419, 0.1011	0.0676, 0.1534	0.0581, 0.1427	0.0707, 0.1596	0.0369, 0.0815
GOOF	1.155	1.106	1.198	1.126	1.126	1.108
*ρ*_min_, *ρ*_max_, *e*/Å^3^	0.72/−1.04	1.29/−0.91	1.73/−1.25	2.22/−1.53	1.88/−1.42	2.31/−4.60
CSD	2048047	2048048	2048049	2048046	2064850	2063823

^1^ The compound **4** was refined using twin law with the creation of an HKLF5-type reflection file. The MERG code was changed to 0 for compatibility with the HKLF and BASF parameters.

## Data Availability

Crystallographic data are available as Supplementary Materials and have been deposited with the Inorganic Crystal Structure Database and can be obtained from Fachinformationszentrum Karlsruhe via www.fiz-karlsruhe.de/request_for_deposited_data.html (accessed on March 24 2021; deposition numbers CSD: 2048046–2048049, 2063823 and 2064850).

## Supplementary Materials

The following are available online: Crystallographic information files of compounds **1**–**6**.

## Sample Availability

Samples of the compounds are available from the authors.

## Appendix A

**Table A1:** Selected geometric parameters for **1**–**5**.

Compound **1**			
Bond distances, Å			
Cu1—Cu2	2.9637 (5)	Cu2—O3	1.8545 (9)
Cu1—O1	1.840 (4)	P1—O1	1.505 (4)
Cu1—O2	2.154 (2)	P1—O2	1.541 (2)
Cu1—O3	1.863 (3)	P1—O2	1.541 (2)
Cu2—O2	2.0251 (19)	P1—O2	1.541 (2)
Angles, °			
Cu2—O3—Cu1	105.72 (10) (×3)	Cu2—O3—Cu2	112.95 (8) (×3)
Compound **2**			
Bond distances, Å			
Cu1—O3	2.066 (3)	Cu4—O3	2.005 (3)
Cu1—O5	1.874 (5)	Cu4—O6	2.003 (3)
Cu1—O7	1.911 (5)	Cu4—O7	1.8747 (10)
Cu2—O1	2.069 (3)	Cu4—O8	1.874 (2)
Cu2—O2	2.061 (3)	V1—O1	1.720 (3)
Cu2—O4	1.876 (3)	V1—O2	1.731 (3)
Cu2—O6	2.066 (3)	V1—O3	1.726 (3)
Cu2—O8	1.906 (3)	V1—O4	1.667 (3)
Cu3—O1^v^	2.004 (3)	V2—O5	1.668 (5)
Cu3—O2	2.001 (3)	V2—O6	1.727 (3)
Cu3—O8	1.875 (2)	V2—O6	1.727 (3)
Cu3—O8	1.871 (2)	V2—O6	1.727 (3)
Angles, °			
Cu4—O7—Cu1^v^	101.10 (13) (×3)	Cu4—O7—Cu4	116.38 (9) (×3)
Cu3—O8—Cu2	101.34 (12)	Cu4—O8—Cu2	101.20 (12)
Cu3—O8—Cu3^v^	116.28 (13)	Cu4—O8—Cu3^v^	115.98 (13)
Cu3—O8—Cu4	116.64 (13)		
Compound **3**			
Bond distances, Å			
Cu1—O1	1.992 (4)	Cu3—O1	1.989 (3)
Cu1—O2	1.974 (4)	Cu3—O1	1.989 (3)
Cu1—O4	1.855 (3)	Cu3—O5	1.875 (3)
Cu1—O5	1.884 (3)	Cu3—O5	1.875 (3)
Cu2—O2	2.036 (3)	P1—O1	1.556 (4)
Cu2—O3^v^	1.981 (4)	P1—O2	1.545 (4)
Cu2—O5	1.869 (3)	P1—O3	1.517 (4)
Cu2—O5	1.900 (3)	P1—O4	1.501 (4)
Angles, °			
Cu1—O5—Cu2^v^	99.56 (14)	Cu2—O5—Cu3	109.90 (16)
Cu2—O5—Cu1	119.56 (17)	Cu3—O5—Cu1	98.30 (14)
Cu2—O5—Cu2^v^	111.65 (16)	Cu3—O5—Cu2^v^	117.36 (17)
Compound **4**			
Bond distances, Å			
Cu1—O1	1.984 (7)	Cu2—O5	1.932 (6)
Cu1—O2	1.953 (7)	Cu3—O1	1.971 (6)
Cu1—O4	1.865 (6)	Cu3—O5	1.895 (6)
Cu1—O5	1.889 (6)	P1—O1	1.554 (7)
Cu2—O2	2.037 (6)	P1—O2	1.551 (7)
Cu2—O3	1.969 (7)	P1—O3	1.522 (8)
Cu2—O5	1.894 (6)	P1—O4	1.510 (6)
Angles, °			
Cu1—O5—Cu2	96.7 (3)	Cu2—O5—Cu2	112.1 (3)
Cu1—O5—Cu2	126.3 (3)	Cu2—O5—Cu3	108.0 (3)
Cu1—O5—Cu3	95.2 (3)	Cu3—O5—Cu2^v^	118.4 (3)
Compound **5**			
Bond distances, Å			
Cu1—O1	1.982 (6)	Cu2—O5	1.884 (5)
Cu1—O2	1.956 (6)	Cu3—O1	1.982 (6)
Cu1—O4	1.866 (5)	Cu3—O5	1.888 (5)
Cu1—O5	1.900 (5)	P1—O1	1.559 (6)
Cu2—O2	2.044 (6)	P1—O2	1.556 (6)
Cu2—O3	1.980 (6)	P1—O3	1.514 (6)
Cu2—O5	1.923 (5)	P1—O4	1.516 (5)
Angles, °			
Cu1—O5—Cu2	97.2 (2)	Cu2—O5—Cu2	111.2 (3)
Cu1—O5—Cu2	124.6 (3)	Cu2—O5—Cu3	108.7 (3)
Cu1—O5—Cu3	96.1 (2)	Cu3—O5—Cu2	119.0 (3)
Compound **6**			
Bond distances, Å			
Cu1—O1	1.889 (1) ×2	Cu2A—O2	1.888 (2)
Cu1—O3	1.979 (3) ×2	Cu2A—O1	1.928 (1)
Cu2—Cu2A	0.25 (5)	Cu2A—O3	1.98 (2) ×2
Cu2—O2	1.852 (2)	Cu2A—O3	2.35 (5)
Cu2—O1	1.930 (2)	V1—O2	1.655 (4)
Cu2—O3	2.098 (4) ×3	V1—O3	1.742 (3) ×3
