# Supplementary material for: Expanding the Averievite Family, (MX)Cu5O2(T5+O4)2 (T5+ = P, V; M = K, Rb, Cs, Cu; X = Cl, Br): Synthesis and Single-Crystal X-ray Diffraction Study
Source: Molecules. 2021 Mar 24;26(7):1833. doi: 10.3390/molecules26071833 (PMC8036531; doi:10.3390/molecules26071833)

checkCIF/PLATON report

Structure factors have been supplied for datablock(s) averievite-VCsCl

THIS REPORT IS FOR GUIDANCE ONLY. IF USED AS PART OF A REVIEW PROCEDURE FOR PUBLICATION, IT SHOULD NOT REPLACE THE EXPERTISE OF AN EXPERIENCED CRYSTALLOGRAPHIC REFEREE.

No syntax errors found. CIF dictionary Interpreting this report

Datablock: averievite-VCsCl

Bond precision: V- O = 0.0039 A Wavelength=0.71073

Cell:	a=12.7249(4)	b=12.7249(4)	c=8.3767(2)
	alpha=90	beta=90	gamma=120
Temperature:	296 K		

	Calculated	Reported
Volume	1174.66(8)	1174.66(8)
Space group	P -3	P -3
Hall group	-P 3	-P 3
Moiety formula	Cu10 O20 V4, 0.75(Cs2), 2(Cl), Cs0.50	0.25(Cl3 Cs4.02 Cu20 O40 V8), 0.25(Cl)
Sum formula	Cl2 Cs2 Cu10 O20 V4	Cl Cs Cu5 O10 V2
Mr	1495.98	748.60
Dx,g cm-3	4.230	4.233
Z	2	4
Mu (mm-1)	13.717	13.733
F000	1372.0	1373.0
F000’	1380.51	
h,k,lmax	16,16,10	16,14,10
Nref	1810	1810
Tmin,Tmax	0.004,0.503	0.329,1.000
Tmin’	0.002	

Correction method= # Reported T Limits: Tmin=0.329 Tmax=1.000
AbsCorr = MULTI-SCAN

Data completeness= 1.000 Theta(max)= 27.467

R(reflections)= 0.0332(1371) wR2(reflections)= 0.1011(1810)

S = 1.106 Npar= 134

```
The following ALERTS were generated. Each ALERT has the format
       test-name_ALERT_alert-type_alert-level.
Click on the hyperlinks for more details of the test.
```

Alert level B

```
PLAT112_ALERT_2_B ADDSYM Detects New (Pseudo) Symm. Elem         m        100 %Fit
```

Author Response: The resulting structure after applying the ADDSYM program is inconsistent with the observed reflection data.

```
PLAT112_ALERT_2_B ADDSYM Detects New (Pseudo) Symm. Elem         m        100 %Fit
```

Author Response: The resulting structure after applying the ADDSYM program is inconsistent with the observed reflection data.

```
PLAT112_ALERT_2_B ADDSYM Detects New (Pseudo) Symm. Elem         m        100 %Fit
```

Author Response: The resulting structure after applying the ADDSYM program is inconsistent with the observed reflection data.

```
PLAT113_ALERT_2_B ADDSYM Suggests Possible Pseudo/New  Space Group        P-1 Check
              Note: (Pseudo) Lattice Translation Implemented
```

Author Response: The resulting structure after applying the ADDSYM program is inconsistent with the observed reflection data.

Alert level C

```
ABSTY02_ALERT_1_C  An _exptl_absorpt_correction_type has been given without
            a literature citation. This should be contained in the
            _exptl_absorpt_process_details field.
            Absorption correction given as multi-scan
PLAT068_ALERT_1_C Reported F000 Differs from Calcd (or Missing)...     Please Check
PLAT242_ALERT_2_C Low    'MainMol' Ueq as Compared to Neighbors of         V1 Check
PLAT250_ALERT_2_C Large U3/U1 Ratio for Average U(i,j) Tensor ....        3.8 Note
PLAT906_ALERT_3_C Large K Value in the Analysis of Variance ......      9.573 Check
PLAT973_ALERT_2_C Check Calcd Positive Resid. Density on      Cs2A       1.26 eA-3
```

Alert level G

```
PLAT004_ALERT_5_G Polymeric Structure Found with Maximum Dimension          1 Info
PLAT042_ALERT_1_G Calc. and Reported MoietyFormula Strings  Differ     Please Check
PLAT045_ALERT_1_G Calculated and Reported Z Differ by a Factor ...       0.50 Check
PLAT110_ALERT_2_G ADDSYM Detects Potential Lattice Translation ...          ? Check
PLAT112_ALERT_2_G ADDSYM Detects New (Pseudo) Symm. Elem       b/2        100 %Fit
```

Author Response: The resulting structure after applying the ADDSYM program is inconsistent with the observed reflection data.

```
PLAT112_ALERT_2_G ADDSYM Detects New (Pseudo) Symm. Elem         C        100 %Fit
```

Author Response: The resulting structure after applying the ADDSYM program is inconsistent with the observed reflection data.

```
PLAT112_ALERT_2_G ADDSYM Detects New (Pseudo) Symm. Elem       a/2        100 %Fit
```

Author Response: The resulting structure after applying the ADDSYM program is inconsistent with the observed reflection data.

```
PLAT300_ALERT_4_G Atom Site Occupancy of Cs1        Constrained at        0.5 Check
PLAT302_ALERT_4_G Anion/Solvent/Minor-Residue Disorder (Resd  2  )       100% Note
PLAT302_ALERT_4_G Anion/Solvent/Minor-Residue Disorder (Resd  3  )       100% Note
PLAT302_ALERT_4_G Anion/Solvent/Minor-Residue Disorder (Resd  6  )       100% Note
PLAT794_ALERT_5_G Tentative Bond Valency for Cu1       (II)      .       2.18 Info
PLAT794_ALERT_5_G Tentative Bond Valency for Cu2       (II)      .       2.18 Info
PLAT794_ALERT_5_G Tentative Bond Valency for V1        (V)       .       5.15 Info
PLAT794_ALERT_5_G Tentative Bond Valency for V2        (V)       .       5.12 Info
PLAT941_ALERT_3_G Average HKL Measurement Multiplicity ...........        3.4 Low
PLAT951_ALERT_5_G Calculated (ThMax) and CIF-Reported Kmax Differ           2 Units
```

```
   0 ALERT level A = Most likely a serious problem - resolve or explain
   4 ALERT level B = A potentially serious problem, consider carefully
   6 ALERT level C = Check. Ensure it is not caused by an omission or oversight
  17 ALERT level G = General information/check it is not something unexpected

   4 ALERT type 1 CIF construction/syntax error, inconsistent or missing data
  11 ALERT type 2 Indicator that the structure model may be wrong or deficient
   2 ALERT type 3 Indicator that the structure quality may be low
   4 ALERT type 4 Improvement, methodology, query or suggestion
   6 ALERT type 5 Informative message, check
```

It is advisable to attempt to resolve as many as possible of the alerts in all categories. Often the minor alerts point to easily fixed oversights, errors and omissions in your CIF or refinement strategy, so attention to these fine details can be worthwhile. In order to resolve some of the more serious problems it may be necessary to carry out additional measurements or structure refinements. However, the purpose of your study may justify the reported deviations and the more serious of these should normally be commented upon in the discussion or experimental section of a paper or in the "special_details" fields of the CIF. checkCIF was carefully designed to identify outliers and unusual parameters, but every test has its limitations and alerts that are not important in a particular case may appear. Conversely, the absence of alerts does not guarantee there are no aspects of the results needing attention. It is up to the individual to critically assess their own results and, if necessary, seek expert advice.

Publication of your CIF in IUCr journals

A basic structural check has been run on your CIF. These basic checks will be run on all CIFs submitted for publication in IUCr journals (*Acta Crystallographica*, *Journal of Applied Crystallography*, *Journal of Synchrotron Radiation*); however, if you intend to submit to *Acta Crystallographica Section C* or *E* or *IUCrData*, you should make sure that full publication checks are run on the final version of your CIF prior to submission.

Publication of your CIF in other journals

Please refer to the *Notes for Authors* of the relevant journal for any special instructions relating to CIF submission.

PLATON version of 18/09/2020; check.def file version of 20/08/2020

Datablock averievite-VCsCl - ellipsoid plot

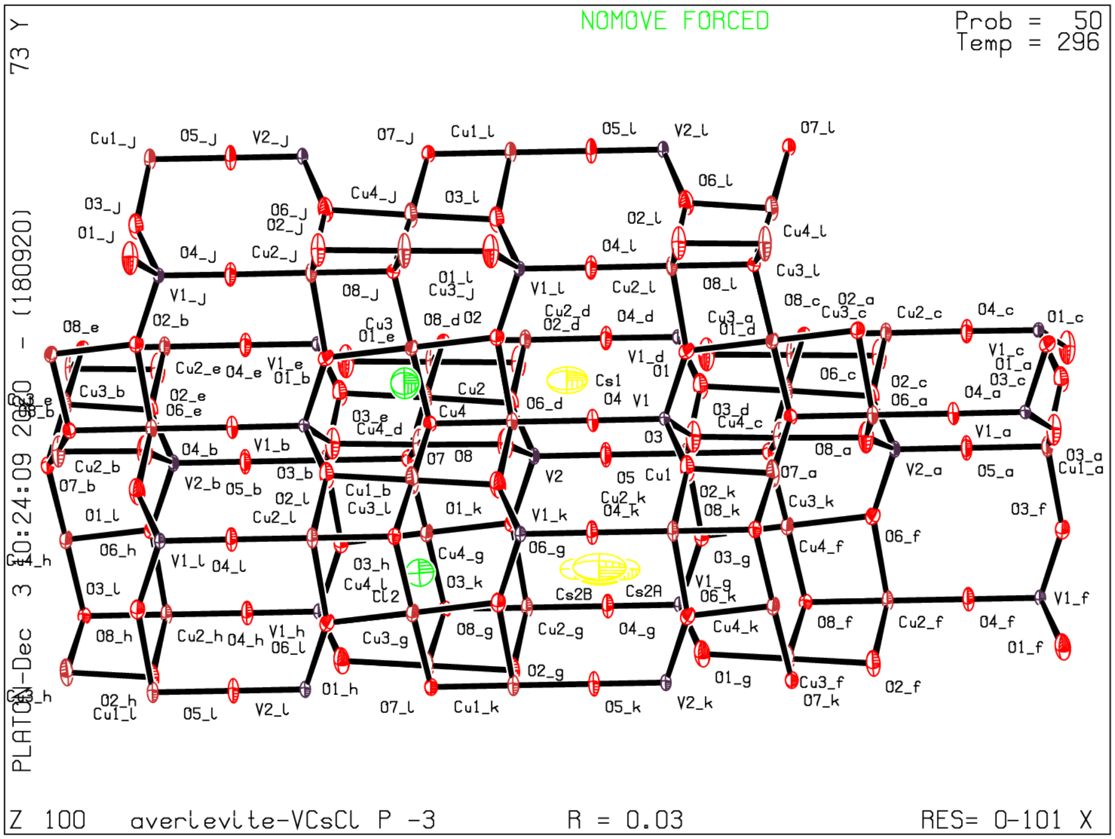

Supplement: Supplementary file 1 [file molecules-26-01833-s001.zip › Compound_2.pdf]
