# Supplementary material for: Expanding the Averievite Family, (MX)Cu5O2(T5+O4)2 (T5+ = P, V; M = K, Rb, Cs, Cu; X = Cl, Br): Synthesis and Single-Crystal X-ray Diffraction Study
Source: Molecules. 2021 Mar 24;26(7):1833. doi: 10.3390/molecules26071833 (PMC8036531; doi:10.3390/molecules26071833)

checkCIF/PLATON report

Structure factors have been supplied for datablock(s) averievite-PKCl

THIS REPORT IS FOR GUIDANCE ONLY. IF USED AS PART OF A REVIEW PROCEDURE FOR PUBLICATION, IT SHOULD NOT REPLACE THE EXPERTISE OF AN EXPERIENCED CRYSTALLOGRAPHIC REFEREE.

No syntax errors found. CIF dictionary Interpreting this report

Datablock: averievite-PKCl

```
Bond precision:  P- O = 0.0060 A         Wavelength=0.71073

Cell:           a=10.9808(10)     b=6.2384(5)       c=15.8684(14)
                alpha=90          beta=95.227(8)    gamma=90
Temperature:    296 K

                Calculated                  Reported
Volume          1082.51(16)                 1082.51(16)
Space group     C 2/c                       C 1 2/c 1
Hall group      -C 2yc                      -C 2yc
Moiety formula  Cl2 Cu10 O20 P4, 2(K)       0.25(Cl4 Cu20 K4 O40 P8)
Sum formula     Cl2 Cu10 K2 O20 P4          Cl Cu5 K O10 P2
Mr              1228.48                     614.19
Dx,g cm-3       3.769                       3.769
Z               2                           4
Mu (mm-1)       10.632                      10.632
F000            1164.0                      1164.0
F000'           1173.06
h,k,lmax        14,8,20                     14,8,20
Nref            1245                        1218
Tmin,Tmax       0.290,0.475                 0.917,1.000
Tmin'           0.217

Correction method= # Reported T Limits: Tmin=0.917 Tmax=1.000
AbsCorr = MULTI-SCAN

Data completeness= 0.978          Theta(max)= 27.497

R(reflections)= 0.0626( 1077)     wR2(reflections)= 0.1596( 1218)

S = 1.126                  Npar= 92
```

The following ALERTS were generated. Each ALERT has the format
test-name_ALERT_alert-type_alert-level.
Click on the hyperlinks for more details of the test.

Alert level C

```
PLAT029_ALERT_3_C _diffrn_measured_fraction_theta_full value Low .      0.978 Why?
PLAT260_ALERT_2_C Large Average Ueq of Residue Including        K1      0.132 Check
PLAT906_ALERT_3_C Large K Value in the Analysis of Variance ......      4.047 Check
PLAT911_ALERT_3_C Missing FCF Refl Between Thmin & STh/L=    0.600         21 Report
```

Alert level G

```
PLAT004_ALERT_5_G Polymeric Structure Found with Maximum Dimension          2 Info
PLAT042_ALERT_1_G Calc. and Reported MoietyFormula Strings  Differ     Please Check
PLAT045_ALERT_1_G Calculated and Reported Z Differ by a Factor ...       0.50 Check
PLAT083_ALERT_2_G SHELXL Second Parameter in WGHT  Unusually Large      15.63 Why ?
PLAT300_ALERT_4_G Atom Site Occupancy of K1         Constrained at        0.5 Check
PLAT302_ALERT_4_G Anion/Solvent/Minor-Residue Disorder (Resd  2  )       100% Note
PLAT794_ALERT_5_G Tentative Bond Valency for Cu3       (II)      .       2.11 Info
PLAT870_ALERT_4_G ALERTS Related to Twinning Effects Suppressed ..          ! Info
PLAT910_ALERT_3_G Missing # of FCF Reflection(s) Below Theta(Min).          1 Note
PLAT912_ALERT_4_G Missing # of FCF Reflections Above STh/L=  0.600          5 Note
PLAT913_ALERT_3_G Missing # of Very Strong Reflections in FCF ....          1 Note
PLAT941_ALERT_3_G Average HKL Measurement Multiplicity ...........        4.4 Low
```

```
   0 ALERT level A = Most likely a serious problem - resolve or explain
   0 ALERT level B = A potentially serious problem, consider carefully
   4 ALERT level C = Check. Ensure it is not caused by an omission or oversight
  12 ALERT level G = General information/check it is not something unexpected

   2 ALERT type 1 CIF construction/syntax error, inconsistent or missing data
   2 ALERT type 2 Indicator that the structure model may be wrong or deficient
   6 ALERT type 3 Indicator that the structure quality may be low
   4 ALERT type 4 Improvement, methodology, query or suggestion
   2 ALERT type 5 Informative message, check
```

It is advisable to attempt to resolve as many as possible of the alerts in all categories. Often the minor alerts point to easily fixed oversights, errors and omissions in your CIF or refinement strategy, so attention to these fine details can be worthwhile. In order to resolve some of the more serious problems it may be necessary to carry out additional measurements or structure refinements. However, the purpose of your study may justify the reported deviations and the more serious of these should normally be commented upon in the discussion or experimental section of a paper or in the "special_details" fields of the CIF. checkCIF was carefully designed to identify outliers and unusual parameters, but every test has its limitations and alerts that are not important in a particular case may appear. Conversely, the absence of alerts does not guarantee there are no aspects of the results needing attention. It is up to the individual to critically assess their own results and, if necessary, seek expert advice.

Publication of your CIF in IUCr journals

A basic structural check has been run on your CIF. These basic checks will be run on all CIFs submitted for publication in IUCr journals (*Acta Crystallographica*, *Journal of Applied Crystallography*, *Journal of Synchrotron Radiation*); however, if you intend to submit to *Acta Crystallographica Section C* or *E* or *IUCrData*, you should make sure that full publication checks are run on the final version of your CIF prior to submission.

Publication of your CIF in other journals

Please refer to the *Notes for Authors* of the relevant journal for any special instructions relating to CIF submission.

PLATON version of 05/12/2020; check.def file version of 05/12/2020

Datablock averievite-PKCl - ellipsoid plot

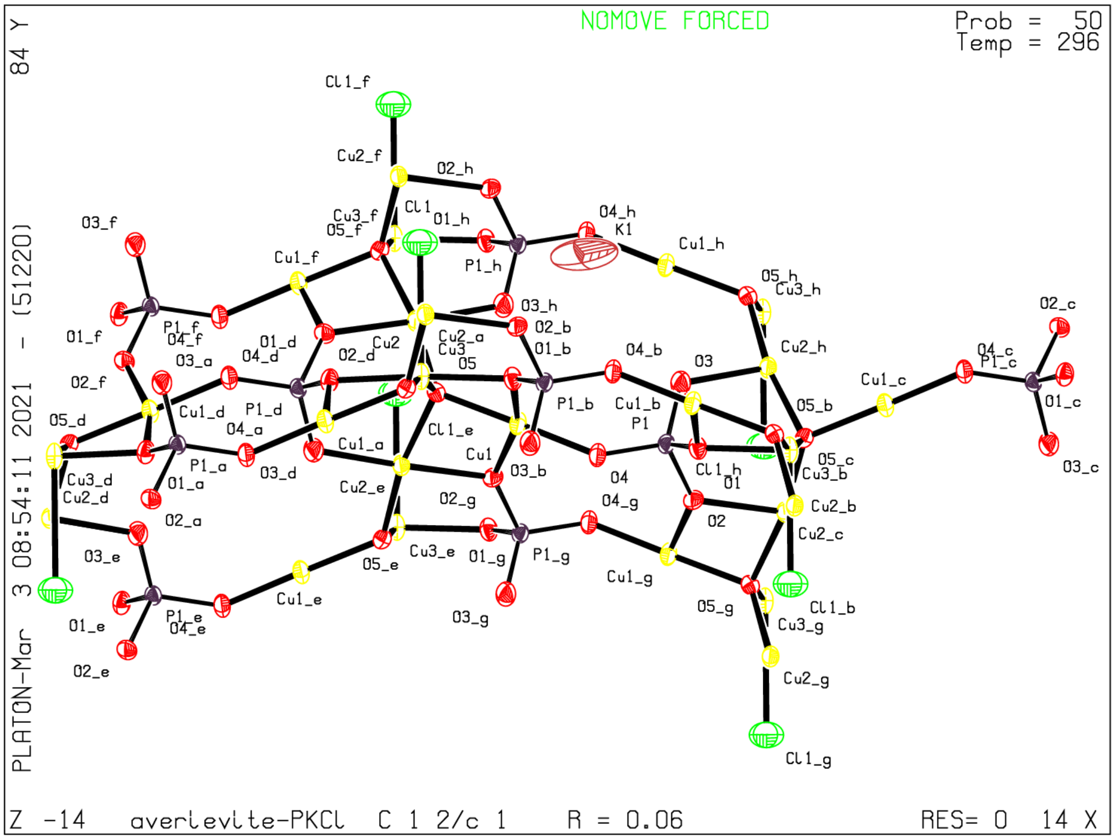

Supplement: Supplementary file 1 [file molecules-26-01833-s001.zip › Compound_5.pdf]
