# Supplementary material for: Expanding the Averievite Family, (MX)Cu5O2(T5+O4)2 (T5+ = P, V; M = K, Rb, Cs, Cu; X = Cl, Br): Synthesis and Single-Crystal X-ray Diffraction Study
Source: Molecules. 2021 Mar 24;26(7):1833. doi: 10.3390/molecules26071833 (PMC8036531; doi:10.3390/molecules26071833)

checkCIF/PLATON report

Structure factors have been supplied for datablock(s) shelx

THIS REPORT IS FOR GUIDANCE ONLY. IF USED AS PART OF A REVIEW PROCEDURE FOR PUBLICATION, IT SHOULD NOT REPLACE THE EXPERTISE OF AN EXPERIENCED CRYSTALLOGRAPHIC REFEREE.

No syntax errors found. CIF dictionary Interpreting this report

Datablock: shelx

```
Bond precision:  V- O = 0.0033 A         Wavelength=0.71073

Cell:               a=6.406(4)       b=6.406(4)       c=8.403(5)
                    alpha=90         beta=90          gamma=120
Temperature:        296 K
```

	Calculated	Reported
Volume	298.6(4)	298.6(4)
Space group	P -3 m 1	P -3 m 1
Hall group	-P 3 2"	-P 3 2"
Moiety formula	Cu4.98 O10 V2, 2.064(Cl), 1.741(Cu)	?
Sum formula	Cl2.06 Cu6.72 O10 V2	Cl2.07 Cu6.74 O10 V2
Mr	762.17	763.52
Dx,g cm-3	4.239	4.246
Z	1	1
Mu (mm-1)	13.710	13.743
F000	356.0	357.0
F000’	359.19	
h,k,lmax	11,11,14	10,10,14
Nref	643	643
Tmin,Tmax		
Tmin’		

```
Correction method= Not given

Data completeness= 1.000          Theta(max)= 37.666

R(reflections)= 0.0326( 578)      wR2(reflections)= 0.0815( 643)

S = 1.108                   Npar= 57
```

The following ALERTS were generated. Each ALERT has the format
test-name_ALERT_alert-type_alert-level.
Click on the hyperlinks for more details of the test.

Alert level A

```
PLAT974_ALERT_2_A Check Calcd Negative Resid. Density on       Cu4      -4.58 eA-3
```

Alert level B

```
DIFMN02_ALERT_2_B  The minimum difference density is < -0.1*ZMAX*1.00
            _refine_diff_density_min given =     -4.600
            Test value =     -2.900
PLAT098_ALERT_2_B Large Reported Min.  (Negative) Residual Density      -4.60 eA-3
PLAT250_ALERT_2_B Large U3/U1 Ratio for Average U(i,j) Tensor ....        4.7 Note
```

Alert level C

```
DIFMN03_ALERT_1_C  The minimum difference density is < -0.1*ZMAX*0.75
            The relevant atom site should be identified.
DIFMX02_ALERT_1_C  The maximum difference density is > 0.1*ZMAX*0.75
            The relevant atom site should be identified.
PLAT041_ALERT_1_C Calc. and Reported SumFormula    Strings  Differ     Please Check
PLAT043_ALERT_1_C Calculated and Reported Mol. Weight Differ by ..       1.35 Check
PLAT052_ALERT_1_C Info on Absorption Correction Method   Not Given     Please Do !
PLAT053_ALERT_1_C Minimum Crystal Dimension Missing (or Error) ...     Please Check
PLAT054_ALERT_1_C Medium  Crystal Dimension Missing (or Error) ...     Please Check
PLAT055_ALERT_1_C Maximum Crystal Dimension Missing (or Error) ...     Please Check
PLAT068_ALERT_1_C Reported F000 Differs from Calcd (or Missing)...     Please Check
PLAT077_ALERT_4_C Unitcell Contains Non-integer Number of Atoms ..     Please Check
PLAT097_ALERT_2_C Large Reported Max.  (Positive) Residual Density       2.31 eA-3
PLAT213_ALERT_2_C Atom Cu2             has ADP max/min Ratio .....        3.1 oblate
PLAT213_ALERT_2_C Atom O3              has ADP max/min Ratio .....        3.1 prolat
PLAT220_ALERT_2_C NonSolvent   Resd 1  O   Ueq(max)/Ueq(min) Range        3.1 Ratio
PLAT260_ALERT_2_C Large Average Ueq of Residue Including       Cu4      0.134 Check
PLAT313_ALERT_2_C Oxygen with Three Covalent Bonds (rare) ........         O2 Check
PLAT927_ALERT_1_C Reported and Calculated  wR2 Differ by .........    -0.0016 Check
PLAT971_ALERT_2_C Check Calcd Resid. Dens.  0.43A   From Cu4             2.04 eA-3
PLAT973_ALERT_2_C Check Calcd Positive Resid. Density on       Cu2       1.43 eA-3
PLAT976_ALERT_2_C Check Calcd Resid. Dens.  0.88A   From O1             -0.47 eA-3
```

Alert level G

```
FORMU01_ALERT_2_G  There is a discrepancy between the atom counts in the
            _chemical_formula_sum and the formula from the _atom_site* data.
            Atom count from _chemical_formula_sum:Cl2.07 Cu6.74 O10 V2
            Atom count from the _atom_site data:  Cl2.064 Cu6.721 O10 V2
PLAT004_ALERT_5_G Polymeric Structure Found with Maximum Dimension          3 Info
PLAT301_ALERT_3_G Main Residue  Disorder .............(Resd  1  )        28% Note
PLAT302_ALERT_4_G Anion/Solvent/Minor-Residue Disorder (Resd  2  )      100% Note
PLAT302_ALERT_4_G Anion/Solvent/Minor-Residue Disorder (Resd  3  )      100% Note
PLAT302_ALERT_4_G Anion/Solvent/Minor-Residue Disorder (Resd  4  )      100% Note
PLAT302_ALERT_4_G Anion/Solvent/Minor-Residue Disorder (Resd  5  )      100% Note
PLAT302_ALERT_4_G Anion/Solvent/Minor-Residue Disorder (Resd  6  )      100% Note
PLAT811_ALERT_5_G No ADDSYM Analysis: Too Many Excluded Atoms ....          ! Info
PLAT883_ALERT_1_G No Info/Value for _atom_sites_solution_primary .     Please Do !
```

1 **ALERT level A** = Most likely a serious problem - resolve or explain
3 **ALERT level B** = A potentially serious problem, consider carefully
20 **ALERT level C** = Check. Ensure it is not caused by an omission or oversight
10 **ALERT level G** = General information/check it is not something unexpected

11 ALERT type 1 CIF construction/syntax error, inconsistent or missing data

```
 14 ALERT type 2 Indicator that the structure model may be wrong or deficient
  1 ALERT type 3 Indicator that the structure quality may be low
  6 ALERT type 4 Improvement, methodology, query or suggestion
  2 ALERT type 5 Informative message, check
```

It is advisable to attempt to resolve as many as possible of the alerts in all categories. Often the minor alerts point to easily fixed oversights, errors and omissions in your CIF or refinement strategy, so attention to these fine details can be worthwhile. In order to resolve some of the more serious problems it may be necessary to carry out additional measurements or structure refinements. However, the purpose of your study may justify the reported deviations and the more serious of these should normally be commented upon in the discussion or experimental section of a paper or in the "special_details" fields of the CIF. checkCIF was carefully designed to identify outliers and unusual parameters, but every test has its limitations and alerts that are not important in a particular case may appear. Conversely, the absence of alerts does not guarantee there are no aspects of the results needing attention. It is up to the individual to critically assess their own results and, if necessary, seek expert advice.

Publication of your CIF in IUCr journals

A basic structural check has been run on your CIF. These basic checks will be run on all CIFs submitted for publication in IUCr journals (*Acta Crystallographica*, *Journal of Applied Crystallography*, *Journal of Synchrotron Radiation*); however, if you intend to submit to *Acta Crystallographica Section C* or *E* or *IUCrData*, you should make sure that full publication checks are run on the final version of your CIF prior to submission.

Publication of your CIF in other journals

Please refer to the *Notes for Authors* of the relevant journal for any special instructions relating to CIF submission.

PLATON version of 05/12/2020; check.def file version of 05/12/2020

Datablock shelx - ellipsoid plot

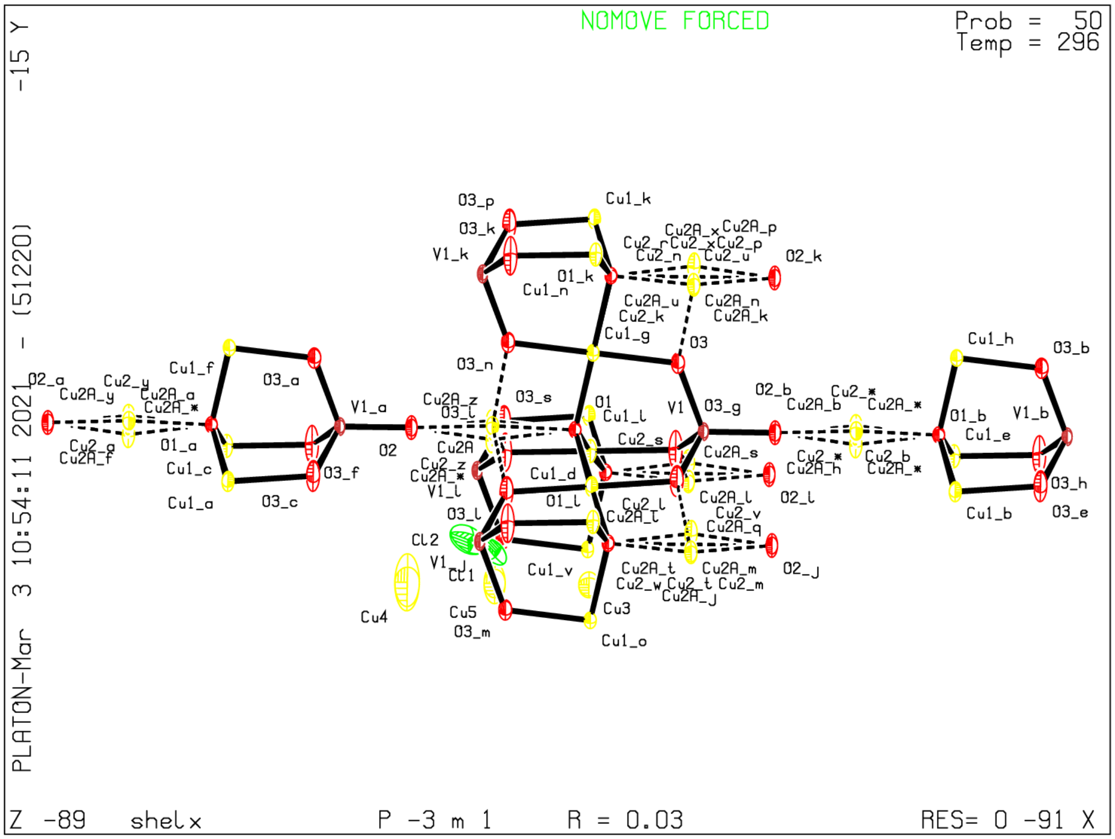

Supplement: Supplementary file 1 [file molecules-26-01833-s001.zip › Compound_6.pdf]
