# Supplementary material for: Expanding the Averievite Family, (MX)Cu5O2(T5+O4)2 (T5+ = P, V; M = K, Rb, Cs, Cu; X = Cl, Br): Synthesis and Single-Crystal X-ray Diffraction Study
Source: Molecules. 2021 Mar 24;26(7):1833. doi: 10.3390/molecules26071833 (PMC8036531; doi:10.3390/molecules26071833)

checkCIF/PLATON report

Structure factors have been supplied for datablock(s) averievite-pcscl

THIS REPORT IS FOR GUIDANCE ONLY. IF USED AS PART OF A REVIEW PROCEDURE FOR PUBLICATION, IT SHOULD NOT REPLACE THE EXPERTISE OF AN EXPERIENCED CRYSTALLOGRAPHIC REFEREE.

No syntax errors found. CIF dictionary Interpreting this report

Datablock: averievite-pcscl

```
Bond precision:  P- O = 0.0030 A         Wavelength=0.71073

Cell:              a=6.1842(1)      b=6.1842(1)      c=8.2333(2)
                   alpha=90         beta=90          gamma=120
Temperature:       296 K
```

	Calculated	Reported
Volume	272.692(11)	272.691(11)
Space group	P -3 m 1	P -3 m 1
Hall group	-P 3 2"	-P 3 2"
Moiety formula	Cu5 O10 P2, Cs0.40, Cl, 0.6(Cs)	2(Cl0.5 Cs0.51 Cu2.5 O5 P1)
Sum formula	Cl Cs Cu5 O10 P2	Cl Cs Cu5 O10 P2
Mr	708.05	708.00
Dx,g cm-3	4.312	4.311
Z	1	1
Mu (mm-1)	13.460	13.460
F000	327.0	327.0
F000’	328.72	
h,k,lmax	8,8,10	8,8,10
Nref	270	269
Tmin,Tmax	0.217,0.668	0.701,1.000
Tmin’	0.182	

```
Correction method= # Reported T Limits: Tmin=0.701 Tmax=1.000
AbsCorr = MULTI-SCAN

Data completeness= 0.996          Theta(max)= 27.499

R(reflections)= 0.0153( 267)      wR2(reflections)= 0.0365( 269)

S = 1.168                   Npar= 33
```

The following ALERTS were generated. Each ALERT has the format
test-name_ALERT_alert-type_alert-level.
Click on the hyperlinks for more details of the test.

Alert level B

```
PLAT250_ALERT_2_B Large U3/U1 Ratio for Average U(i,j) Tensor ....        6.5 Note
```

Author Response: The large U3/U1 ratio is a characteristic of the particular structure.

Alert level C

```
PLAT088_ALERT_3_C Poor Data / Parameter Ratio ....................       8.15 Note
PLAT213_ALERT_2_C Atom Cu1             has ADP max/min Ratio .....        3.2 oblate
PLAT213_ALERT_2_C Atom Cu2             has ADP max/min Ratio .....        3.2 prolat
PLAT743_ALERT_1_C Torsion Calc  -120.01(8), Rep     -120.00 ......    Missing s.u.
              O2  -CU1 -O3  -CU2    9.666   1.555   1.555   2.665  #  121 Check
PLAT753_ALERT_4_C Torsion Calc     -120.00, Rep     -120.00 ......  Senseless s.u.
              CU2 -CU1 -O3  -CU2    2.665   1.555   1.555   1.555  #  109 Check
PLAT753_ALERT_4_C Torsion Calc      120.00, Rep      120.00 ......  Senseless s.u.
              CU2 -CU1 -O3  -CU2    3.565   1.555   1.555   1.555  #  110 Check
PLAT753_ALERT_4_C Torsion Calc     -120.00, Rep     -120.00 ......  Senseless s.u.
              CU2 -CU1 -O3  -CU2    3.565   1.555   1.555   2.665  #  111 Check
PLAT753_ALERT_4_C Torsion Calc      120.00, Rep      120.00 ......  Senseless s.u.
              CU2 -CU1 -O3  -CU2    2.665   1.555   1.555   3.565  #  112 Check
PLAT927_ALERT_1_C Reported and Calculated  wR2 Differ by .........    -0.0021 Check
PLAT973_ALERT_2_C Check Calcd Positive Resid. Density on      Cs1A       1.14 eA-3
```

Alert level G

```
FORMU01_ALERT_1_G  There is a discrepancy between the atom counts in the
            _chemical_formula_sum and _chemical_formula_moiety. This is
            usually due to the moiety formula being in the wrong format.
            Atom count from _chemical_formula_sum:   Cl1 Cs1 Cu5 O10 P2
            Atom count from _chemical_formula_moiety:Cl1 Cs1.02 Cu5 O10 P2
PLAT004_ALERT_5_G Polymeric Structure Found with Maximum Dimension          3 Info
PLAT042_ALERT_1_G Calc. and Reported MoietyFormula Strings  Differ     Please Check
PLAT302_ALERT_4_G Anion/Solvent/Minor-Residue Disorder (Resd  2  )       100% Note
PLAT302_ALERT_4_G Anion/Solvent/Minor-Residue Disorder (Resd  4  )       100% Note
PLAT794_ALERT_5_G Tentative Bond Valency for Cu1       (II)      .       2.09 Info
PLAT883_ALERT_1_G No Info/Value for _atom_sites_solution_primary .     Please Do !
PLAT910_ALERT_3_G Missing # of FCF Reflection(s) Below Theta(Min).          1 Note
PLAT961_ALERT_5_G Dataset Contains no Negative Intensities .......     Please Check
```

```
   0 ALERT level A = Most likely a serious problem - resolve or explain
   1 ALERT level B = A potentially serious problem, consider carefully
  10 ALERT level C = Check. Ensure it is not caused by an omission or oversight
   9 ALERT level G = General information/check it is not something unexpected

   5 ALERT type 1 CIF construction/syntax error, inconsistent or missing data
   4 ALERT type 2 Indicator that the structure model may be wrong or deficient
   2 ALERT type 3 Indicator that the structure quality may be low
   6 ALERT type 4 Improvement, methodology, query or suggestion
   3 ALERT type 5 Informative message, check
```

It is advisable to attempt to resolve as many as possible of the alerts in all categories. Often the minor alerts point to easily fixed oversights, errors and omissions in your CIF or refinement strategy, so attention to these fine details can be worthwhile. In order to resolve some of the more serious problems it may be necessary to carry out additional measurements or structure refinements. However, the purpose of your study may justify the reported deviations and the more serious of these should normally be commented upon in the discussion or experimental section of a paper or in the "special_details" fields of the CIF. checkCIF was carefully designed to identify outliers and unusual parameters, but every test has its limitations and alerts that are not important in a particular case may appear. Conversely, the absence of alerts does not guarantee there are no aspects of the results needing attention. It is up to the individual to critically assess their own results and, if necessary, seek expert advice.

Publication of your CIF in IUCr journals

A basic structural check has been run on your CIF. These basic checks will be run on all CIFs submitted for publication in IUCr journals (*Acta Crystallographica*, *Journal of Applied Crystallography*, *Journal of Synchrotron Radiation*); however, if you intend to submit to *Acta Crystallographica Section C* or *E* or *IUCrData*, you should make sure that full publication checks are run on the final version of your CIF prior to submission.

Publication of your CIF in other journals

Please refer to the *Notes for Authors* of the relevant journal for any special instructions relating to CIF submission.

PLATON version of 18/09/2020; check.def file version of 20/08/2020

Datablock averievite-pcscl - ellipsoid plot

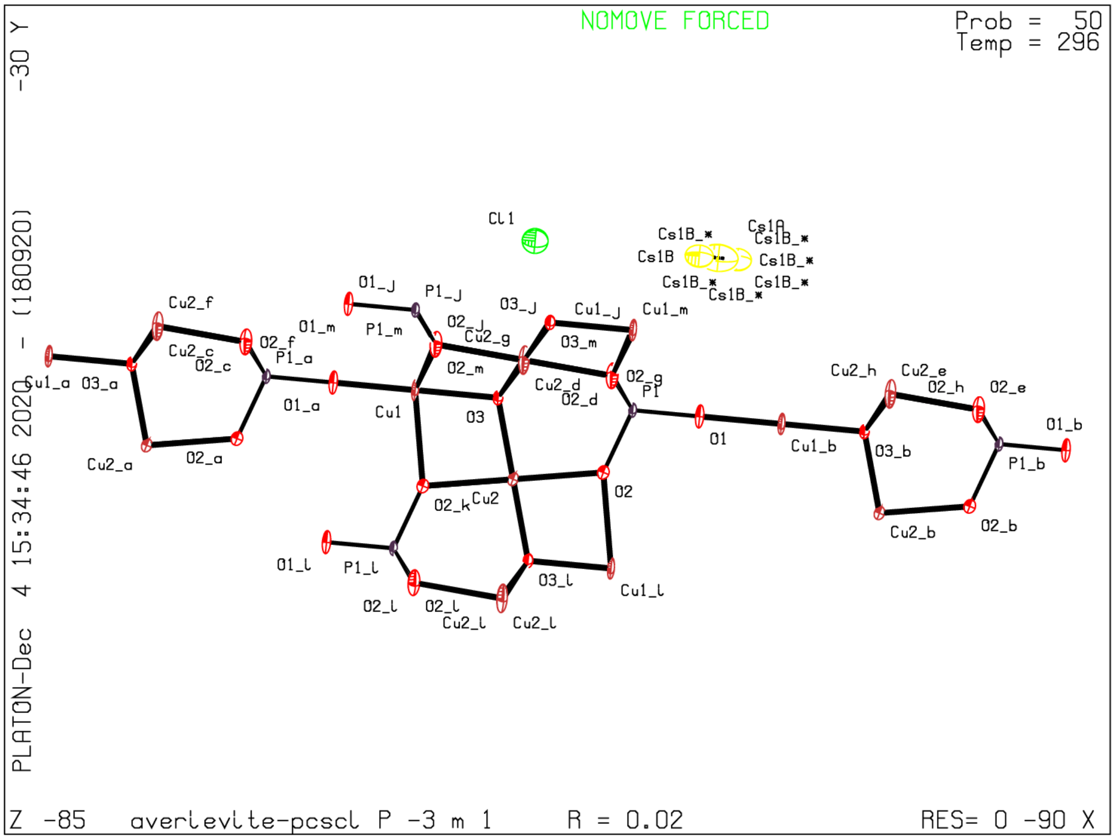

Supplement: Supplementary file 1 [file Compound_1.pdf › Compound_1.pdf]
